# The epidemiology and prognosis of patients with primary gastric T‐cell lymphoma in the SEER program

**DOI:** 10.1002/cam4.4936

**Published:** 2022-06-13

**Authors:** Minyue Zhang, Fei Xiao, Meisi Lin, Mengping Chen, Jian Hou, Honghui Huang

**Affiliations:** ^1^ Division of Hematology, Renji Hospital, School of Medicine Shanghai Jiaotong University Shanghai China; ^2^ State Key Laboratory of Southwestern Chinese Medicine Resources, Pharmacy School Chengdu University of Traditional Chinese Medicine Chengdu China; ^3^ Sichuan Provincial Acupuncture School Chengdu China

**Keywords:** incidence, primary gastric T‐cell lymphoma, prognosis, SEER

## Abstract

**Background:**

Primary gastric T‐cell lymphoma (PG‐TCL) is a rare hematological malignancy with few data reported. The objective of this study is to investigate the epidemiology, clinical characteristics, and survivals of PG‐TCL.

**Methods:**

Totally, 164 patients with PG‐TCL from 1975 to 2016 extracted from the Surveillance, Epidemiology, and End Results Program (SEER) database were analyzed. Kaplan–Meier method was applied to plot overall survival (OS) and cancer‐specific survival (CSS). The prognostic factors of OS and CSS were explored by Cox proportional hazard regression. Nomograms were constructed to predict survival possibilities.

**Results:**

The age‐adjusted incidence rate of PG‐TCL was 0.0091 per 100,000 person‐years and increased with age. The median age at onset was 65 years old with male predominance. The major histological type was peripheral T‐cell lymphoma, NOS (63.4%). The 1‐, 2‐, and 5‐year OS were 45.5%, 34.7%, and 23.5%, respectively while the 1‐, 2‐, and 5‐year CSS were 47.4%, 37.3%, and 29.6%, respectively. Multivariate Cox analysis demonstrated that age at diagnosis, use of chemotherapy, and radiotherapy were the independent prognostic factors for OS. Chemotherapy combined with radiotherapy could significantly improve patients' OS compared with chemotherapy alone. Moreover, age at diagnosis and use of chemotherapy were also the independent prognostic factors for CSS. Nomograms for PG‐TCL were developed to predict 1‐, 2‐, and 5‐year OS possibilities. The predictability of nomograms was verified by high concordance index and good agreement with the predicted value in calibration plots.

**Conclusion:**

PG‐TCL is a rare neoplasm with low incidence. Patients with PG‐TCL generally exhibited poor prognosis. Use of chemotherapy plus radiotherapy was associated with favorable OS.

## Graphical abstract

This is the first and largest population‐based study about primary gastric T‐cell lymphoma(PG‐TCL). Patients with PG‐TCL generally exhibited poor prognosis. Use of Chemotherapy plus radiotherapy was associated with favorable survival.

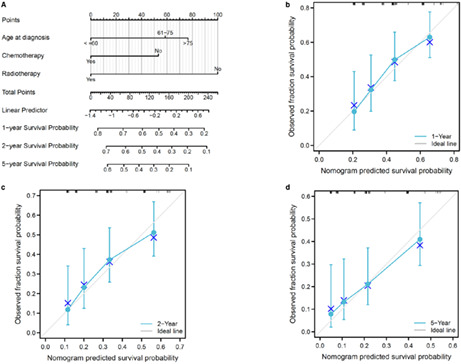

## 1 INTRODUCTION

Primary gastrointestinal lymphomas were firstly described in 1961 by Dawson, et al. as the presence of a predominant gastrointestinal lesion, with or without regional lymphadenopathy but without the involvement of distal lymph nodes, bone marrow, spleen or liver.^1^ This definition was later widened, that is, involving the adjacent liver and spleen, or associated with a smaller nodal component that must represent less than 25% of total tumor volume.^2^, ^3^, ^4^ Primary gastric lymphoma (PGL) is the most common type of extranodal lymphoma, accounting for 30% ~ 40% of all extra‐nodal lymphoma and 55% ~ 65% of all gastrointestinal lymphomas,^5^, ^6^ although it is a rare type of primary gastric neoplasms.^5^ The large majority of PGL cases are diffuse large B‐cell lymphoma (DLBCL), followed by mucosa‐associated lymphoma tissue (MALT) lymphoma.^7^, ^8^, ^9^, ^10^

Nevertheless, primary gastric T‐cell lymphoma (PG‐TCL) is a rare entity with a handful of sporadic case reports or small retrospective series studies in the literature.^11^, ^12^, ^13^, ^14^, ^15^, ^16^, ^17^ The incidence of PG‐TCL was reported to range from 1.9% to 7.0% among all PGL.^13^, ^14^, ^15^ The most common histological subtype of PG‐TCL is peripheral T‐cell lymphoma, not otherwise specified (PTCL‐NOS).^14^ Some PG‐TCL cases were found to be associated with human T‐cell leukemia virus type 1 infection in Japan.^13^ Concerning to survival, the 5‐year overall survival (OS) of PG‐TCL was estimated to be 55% in Kawamoto's study^16^ whereas the median OS of PG‐TCL in Park's cohort was only 10 months.^14^ Due to its extreme rarity, the clinicopathological entity and prognosis of PG‐TCL have not been well characterized so far.

The National Cancer Institute's Surveillance, Epidemiology, and End Results (SEER) program provided patients' information on cancer statistics in United States (US) population. Researchers can utilize this database to investigate incidence, clinical characteristics, survival, and therapy of rare cancers.^17^, ^18^, ^19^ Here, we comprehensively explored the epidemiology and clinical outcomes of PG‐TCL with the application of population‐based data from the SEER database. Besides, a predictive nomogram for survival was established to assess the OS of individual patient with PG‐TCL.

## 2 METHODS

### 2.1 Cohort selection and data collection

The demographic characteristics, treatment information, and survival of patients diagnosed as PG‐TCL between 1975 and 2016 came from the SEER 18 registry database [Incidence‐SEER 18 Regs Custom Data (with additional treatment fields), Nov 2018 Sub (1975–2016 varying)]. The SEER 18 database [Incidence‐SEER Research Data, 18 registries, Nov 2020 Sub (2020–2018)] was available for extracting data of disease incidence which were registered in and after 2000. The International Classification of Diseases for Oncology, Third Edition 3 (ICD‐O‐3) histology codes 9700/3, 9701/3, 9702/3, 9705/3, 9708/3, 9709/3, 9714/3, 9716/3, 9717/3, 9719/3, 9724/3 and 9725/3, along with the primary site of stomach were used to identify as PG‐TCL. The primary site was categorized into four subgroups according to tumor location: upper third of stomach [including Cardia, NOS (C16.0) and Fundus of stomach (16.1)], mid and low of stomach [including Body of stomach (C16.2), Gastric antrum (C16.3), Pylorus (C16.4), Lesser curvature of stomach NOS (C16.5) and Greater curvature of stomach NOS (16.6)], overlapping stomach (C16.8) and stomach NOS (C16.9). Other histological types of lymphoma with stomach as the primary site or non‐gastric T‐cell lymphoma were regarded as comparative cohorts of PG‐TCL.

Baseline characteristics collected from SEER 18 database included age, sex, race, type of disease histology, Ann Arbor stage, lesion location, treatment (use of chemotherapy, radiotherapy, or surgery), cause‐specific death classification, survival months, and vital status. All the SEER data were obtained from the public database.

### 2.2 Statistical analysis

The difference of baseline characteristics was explored by Student's *t*‐test or Wilcoxon test for continuous variables whereas χ^2^ test for categorical variables. The log‐rank test was used to compare OS or cancer‐specific survival (CSS) between different groups and Kaplan–Meier method was applied to plot the survival curve. CSS was defined as the time from diagnosis to death due to PG‐TCL or the last follow‐up. The Cox hazard proportional regression were performed to identify independent prognostic factors of OS or CSS. Variable exhibiting *P* < 0.1 in the univariate analysis was considered as a candidate to perform the subsequent multivariate analysis. Moreover, a nomogram model was established to predict the 1‐, 2‐, and 5‐year OS probabilities for PG‐TCL patients. Concordance index (C‐index) and calibration plot were utilized to assess the accuracy of nomogram model. Statistical analyses were conducted by SPSS 25.0 and R package 3.6.2. All P‐values were bilateral and *P* < 0.05 was considered as a statistical difference.

## 3 RESULTS

### 3.1 Incidence of PG‐TCL

**FIGURE 1:**
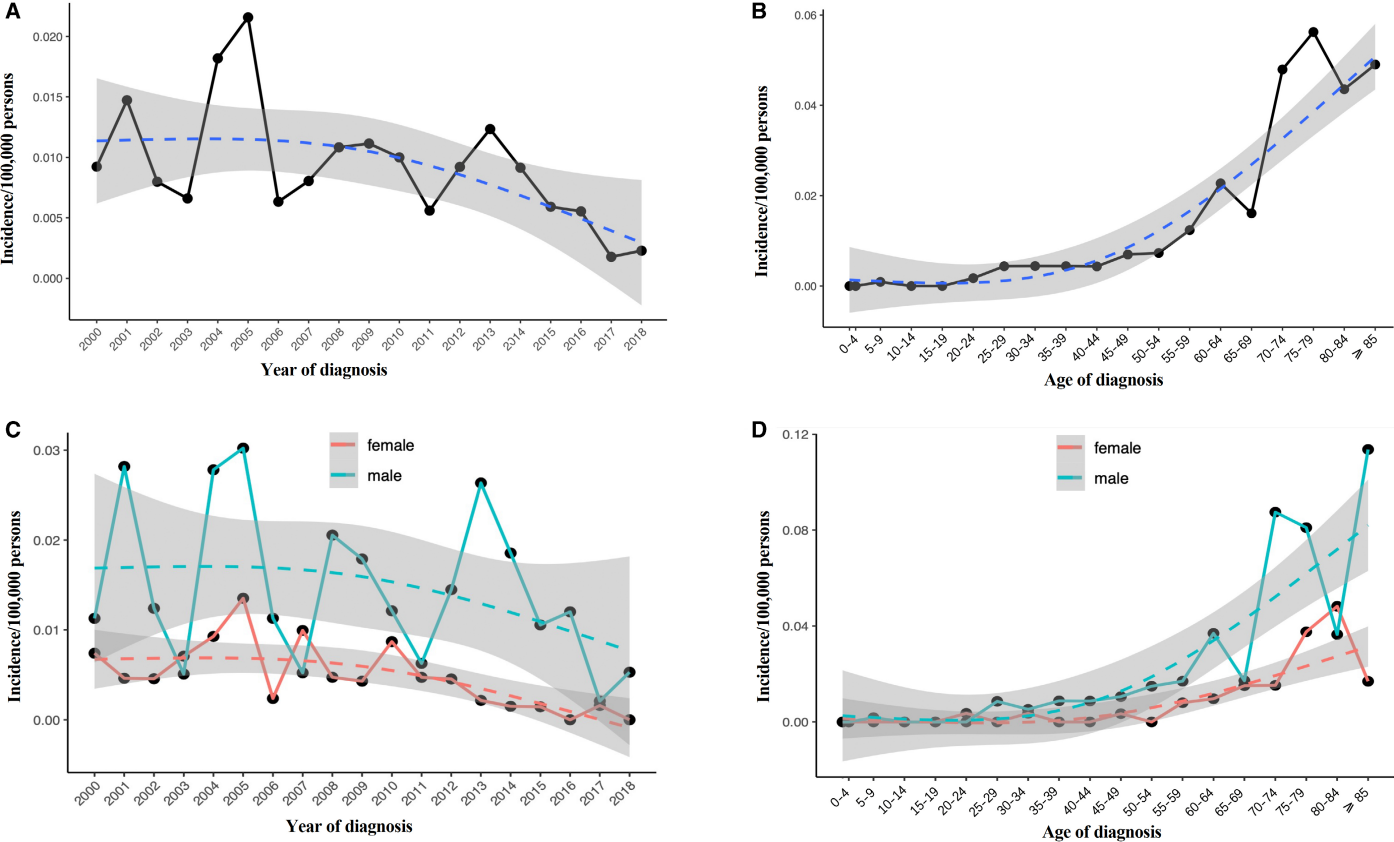
Age‐adjusted incidence of primary gastric T‐cell lymphoma (PG‐TCL) from 2000 to 2018 in SEER database. (A) Annual age‐adjusted incidence of PG‐TCL; (B) Age‐adjusted incidence of PG‐TCL based on age of diagnosis; (C) Annual age‐adjusted incidence of PG‐TCL in male and female populations, respectively; (D) Age‐adjusted incidence of PG‐TCL based on age of diagnosis in male and female populations, respectively.

The SEER database demonstrated that the age‐adjusted incidence rate (AIR) of PG‐TCL from 2000 to 2018 [age adjusted to the 2000 US Standard Population (Census P25‐1130)] was 0.0091 per 100,000 person‐years (95% CI, 0.0076–0.0117). The annual AIR of PG‐TCL was shown in Figure 1A. The AIR was highest in 2005 with 0.0216 per 100,000 person‐years (95% CI, 0.0125–0.0346) and has decreased in the recent 10 years. Furthermore, it is remarkable that the AIR of PG‐TCL increased with age (Figure 1B). Compared with the AIR of patients <60 years old (0.0037/100,000 person‐years), the incidence rate ratio (IRR) for age group 60–69 (AIR 0.0196/100,000 person‐years) and ≥ 70 (AIR 0.497/100,000 person‐years) were 5.37 and 13.61, respectively (P < 0.0001). With regards to gender, the AIR of male (0.0144/100,000 person‐years) was significantly higher than that of female (0.0046/100,000 person‐years, IRR 3.10, 95% CI, 2.13–4.57, *P* < 0.0001, Figure 1C,D).

### 3.2 Demographics of PG‐TCL

The current study identified 164 patients with PG‐TCL from SEER database. The demographics and treatment information of PG‐TCL were shown in Table 1. The median age at diagnosis was 65 years old. Most patients were White (63.4%) and male (68.9%). With regards to tumor sites, 27 (16.5%) patients showed the involvement of the upper third of the stomach, 47 (28.7%) patients had tumors involved in the mid and low of stomach, 17 (10.4%) patients demonstrated the involvement of whole stomach, and the specific primary site of stomach was not recorded for 73 (44.5%) patients. The major histological type was PTCL‐NOS (63.4%), followed by anaplastic large T‐cell lymphoma, anaplastic lymphoma kinase + (ALCL‐ALK+, 31.7%). About half of the patients (51.7%) had Ann Arbor stage I/II. Of 164 patients, 67 (40.9%) only underwent chemotherapy, 4 (2.4%) only received radiotherapy and 8 (4.9%) only underwent surgery. Combined treatment was provided to 27 patients (16.4%). No treatment or no therapy record was found in 58 patients (35.4%).

**TABLE 1:** Demographic and clinical characteristics of primary gastric T‐cell lymphoma patients

Characteristic	Primary gastric T‐cell lymphoma (*N* = 164)	
	Number	% of patients
Age at diagnosis (years old)		
Mean ± SD	63.35 ± 17.64	
Median (range)	65 (8 ~ 93)	
≤60	64	39.1
61 ~ 75	44	26.8
>75	56	34.1
Sex		
Male	113	68.9
Female	51	31.1
Race		
White	104	63.4
Black/Other	59	36.0
Unknown	1	0.6
Primary site		
Upper third of stomach	27	16.5
Mid and low of stomach	47	28.7
Overlapping lesion of stomach	17	10.4
Stomach, NOS	73	44.5
Histology type		
ALCL‐ALK+	52	31.7
PTCL‐NOS	104	63.4
Other T‐NHL	8	4.9
Ann Arbor stage		
I/II	85	51.8
III/IV	58	34.4
Unknown	21	12.8
Symptom		
A	47	28.7
B	40	24.4
Unknown	77	46.9
Chemotherapy		
Yes	93	**56.7**
No/unknown	71	43.3
Radiotherapy		
Yes	17	10.4
No/unknown	147	89.6
Surgery		
Yes	23	14.0
No/unknown	141	86.0
Treatment modality		
No treatment/unknown	58	35.4
Chemotherapy only	67	40.9
Surgery only	8	4.9
Radiotherapy only	4	2.4
Chemotherapy plus Surgery	14	8.5
Chemotherapy plus Radiotherapy	12	7.3
Surgery plus Radiotherapy	1	0.6

Abbreviations: ALCL, anaplastic large cell lymphoma; PTCL, peripheral T‐cell lymphoma.

Subgroup analysis was conducted according to the histological types (PTCL‐NOS, ALCL‐ALK+ and other subtypes of T‐NHL), which was demonstrated in Table S1. There were no significant differences of demographics and clinical characteristics among these three groups of patients.

### 3.3 Survival analysis for PG‐TCL

**FIGURE 2:**
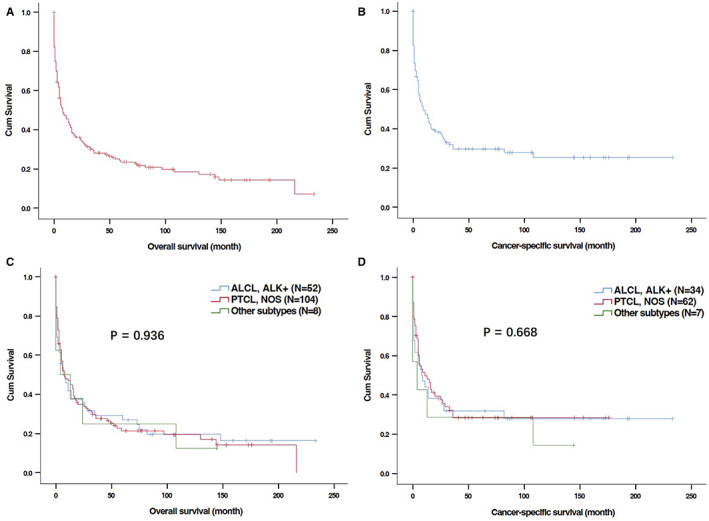
Kaplan–Meier curves for overall survival (OS) and cancer‐specific survival (CSS) of patients with primary gastric T‐cell lymphoma (PG‐TCL) in SEER database. (A) OS of total patients with PG‐TCL; (B) CSS of total patients with PG‐TCL; (C) OS of patients with PG‐TCL according to histological types; (D) CSS of patients with PG‐TCL according to histological types.

With a median follow‐up of 7 (0–223) months, 130 (79.3%) deaths were recorded, and 72 cases died of PG‐TCL. The Kaplan–Meier curves of OS and CSS for total PG‐TCL patents were shown in Figure 2A,B. The median OS and CSS were 8.0 months and 9.0 months, respectively. The 1‐, 2‐, and 5‐year OS were 45.5%, 34.7%, and 23.5%, respectively. The 1‐, 2‐, and 5‐year CSS were 47.4%, 37.3%, and 29.6%, respectively. Regarding histological type of PG‐TCL, no significant difference of OS (Figure 2C, *P* = 0.936) or CSS (Figure 2D, *P* = 0.668) was observed among different subtypes of PG‐TCL patients.

**FIGURE 3:**
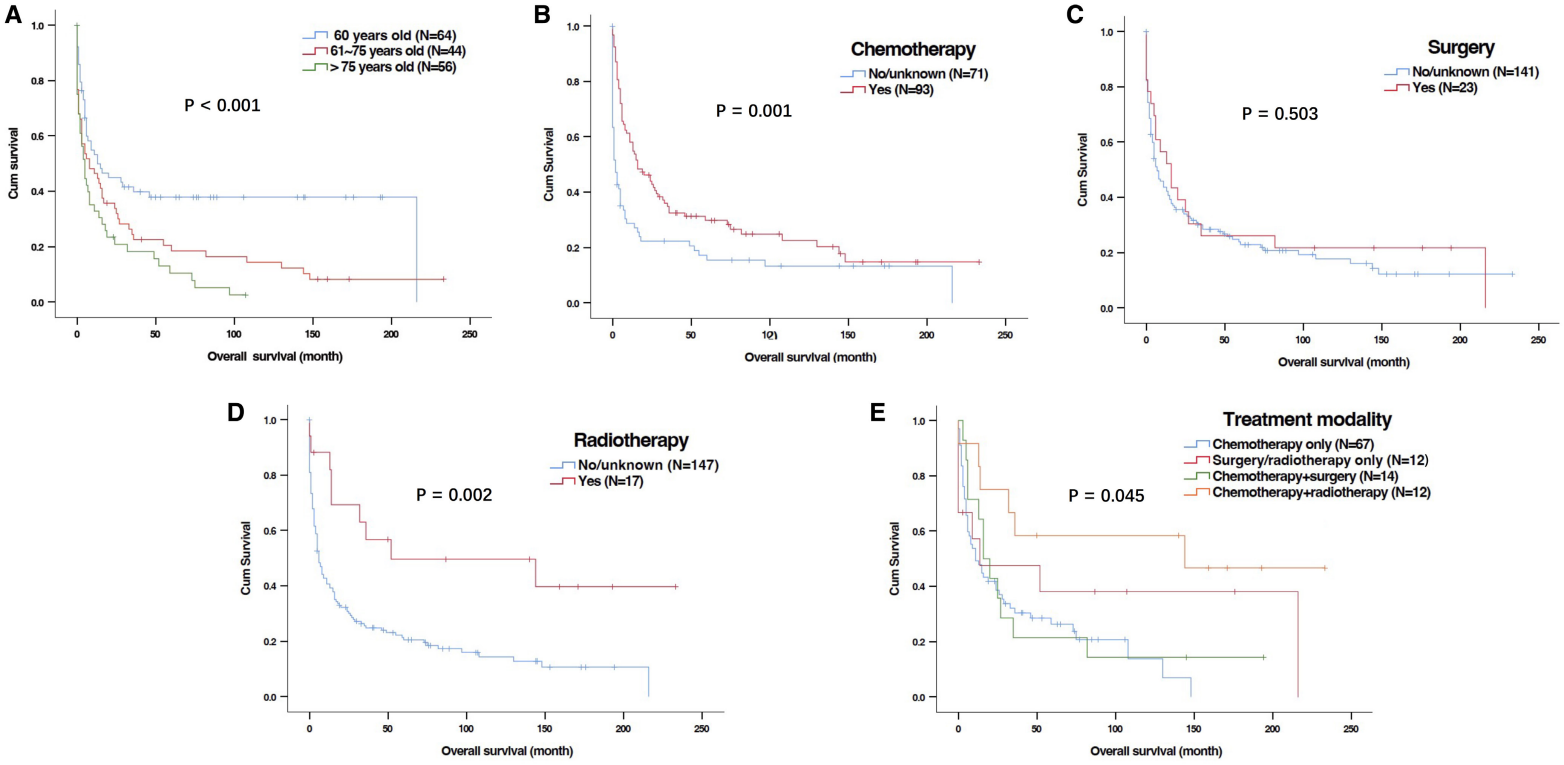
Kaplan–Meier curves for overall survival (OS) of patients with primary gastric T‐cell lymphoma (PG‐TCL) in SEER database based on ages and treatment. (A) Age at diagnosis; (B) Chemotherapy; (C) Surgery; (D) Radiotherapy; (E) Treatment modalities.

**FIGURE 4:**
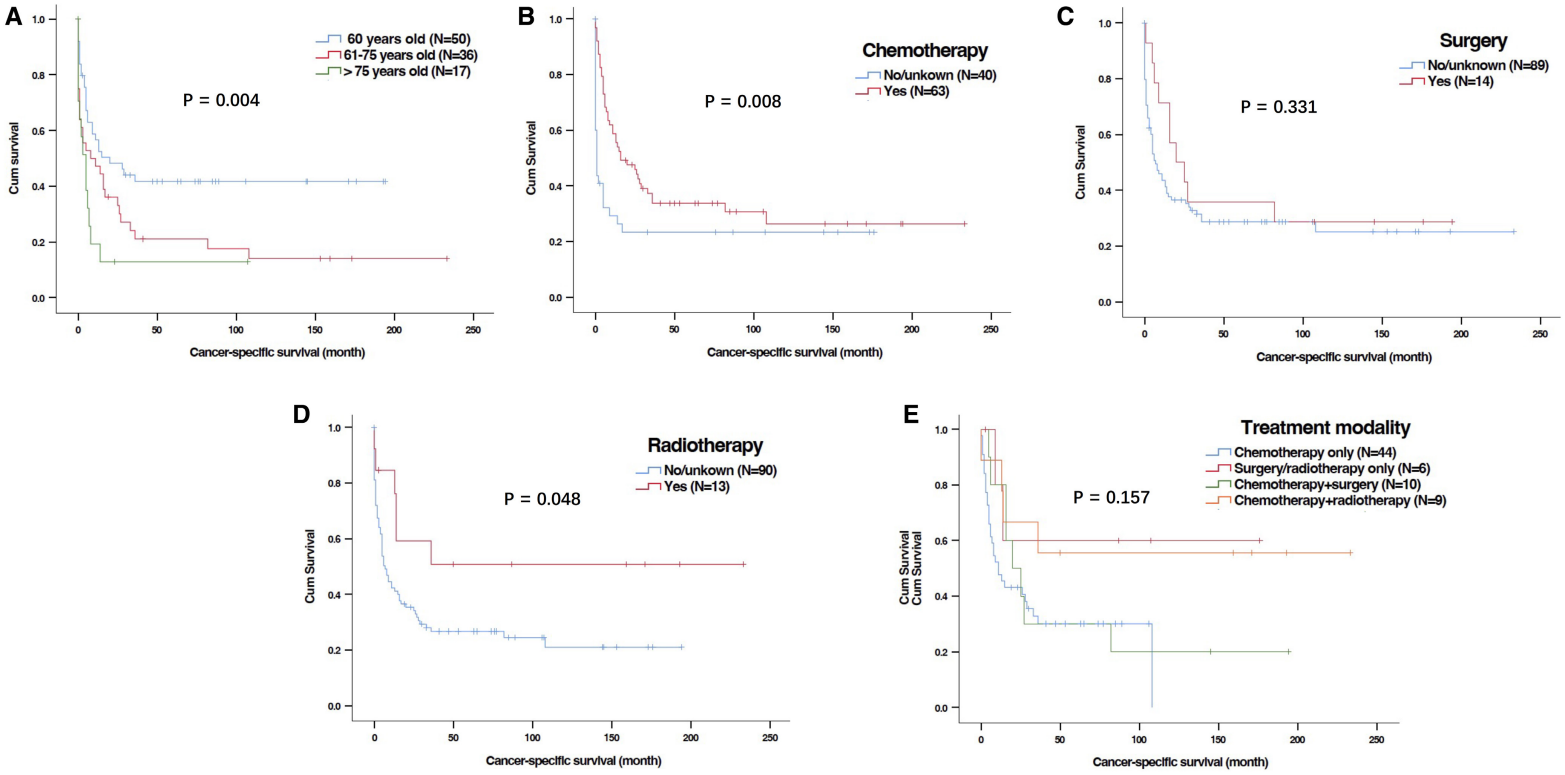
Kaplan–Meier curves for cancer‐specific survival (CSS) of patients with primary gastric T‐cell lymphoma (PG‐TCL) in SEER database based on ages and treatment. (A) Age at diagnosis; (B) Chemotherapy; (C) Surgery; (D) Radiotherapy; (E) Treatment modalities.

Univariate analysis showed that younger age at diagnosis as well as the use of chemotherapy and radiotherapy were obviously associated with better OS (Table 2 and Figure 3A–D) and CSS (Table 3 and Figure 4A–D). In the multivariable analyses, age at diagnosis and chemotherapy were independent predictors for both OS (Table 2 multivariate analysis I) and CSS (Table 3). Radiotherapy had independently prognostic value for OS (Table 2, multivariate analysis I) but a trend of prognostic value for CSS (Table 3).

**TABLE 2:** Univariate and multivariate analyses of overall survival in primary gastric T‐NHL patients (*N* = 164)

Variable	Univariate analysis	*P* value	Multivariate analysis I	*P* value	Multivariate analysis II	*P* value
	HR (95% CI)		HR (95% CI)		HR (95% CI)	
Age at diagnosis (years old)		0.001		0.009		0.026
≤ 60	Reference		Reference		Reference	
61 ~ 75	1.695 (1.114 ~ 2.578)	0.014	1.664 (1.093 ~ 2.532)	0.018	1.910 (1.138 ~ 3.203)	0.014
> 75	2.272 (1.458 ~ 3.539)	< 0.001	1.975 (1.251 ~ 3.116)	0.003	1.888 (1.016 ~ 3.509)	0.044
Sex (Male vs. Female)	0.944 (0.652 ~ 1.366)	0.759				
Race (White vs. Black/Other)	1.227 (0.854 ~ 1.761)	0.269				
Histology type		0.940				
ALCL‐ALK+	Reference					
PTCL‐NOS	1.024 (0.703 ~ 1.491)	0.903				
Other T‐NHL	1.155 (0.517 ~ 2.578)	0.725				
Primary site		0.463				
Upper third of stomach	Reference					
Mid and low of stomach	1.551 (0.890 ~ 2.704)	0.121				
Overlapping lesion of stomach	1.351 (0.669 ~ 2.727)	0.401				
Stomach, NOS	1.457 (0.857 ~ 2.477)	0.165				
Ann Arbor stage (I/II vs. III/IV)	1.051 (0.872 ~ 1.268)	0.600				
Symptom (A vs. B)	1.193 (0.736 ~ 1.935)	0.474				
Chemotherapy (No/unknown vs. Yes)	0.559 (0.394 ~ 0.793)	0.001	0.653 (0.453 ~ 0.939)	0.022		
Radiotherapy (No/unknown vs. Yes)	0.371 (0.187 ~ 0.735)	0.004	0.411 (0.207 ~ 0.816)	0.011		
Surgery (No/unknown vs. Yes)	0.851 (0.521 ~ 1.388)	0.581				
Treatment modality		0.064				0.069
Chemotherapy only	Reference				Reference	
Surgery/Radiotherapy only	0.664 (0.306 ~ 1.442)	0.301			0.736 (0.333 ~ 1.628)	0.450
Chemotherapy plus Surgery	0.856 (0.456 ~ 1.609)	0.629			0.841 (0.440 ~ 1.606)	0.600
Chemotherapy plus Radiotherapy	0.311 (0.130 ~ 0.743)	0.009			0.306 (0.127 ~ 0.739)	0.008

Abbreviations: ALCL, anaplastic large cell lymphoma; PTCL, peripheral T‐cell lymphoma.

**TABLE 3:** Univariate and multivariate analyses of cancer‐specific survival (CSS) in primary gastric T‐cell lymphoma patients (N = 103)

Variable	Univariate analysis	*P* value	Multivariate analysis	*P* value
	HR (95% CI)		HR (95% CI)	
Age at diagnosis (years old)		0.008		0.031
≤ 60	Reference		Reference	
61 ~ 75	1.786 (1.066 ~ 2.994)	0.028	1.725 (1.028 ~ 2.896)	0.039
> 75	2.637 (1.370 ~ 5.075)	0.004	2.220 (1.137 ~ 4.334)	0.019
Sex (Male vs. Female)	0.877 (0.519 ~ 1.484)	0.625		
Race (White vs. Black/Other)	1.033 (0.638 ~ 1.673)	0.895		
Histology type		0.691		
ALCL‐ALK+	Reference			
PTCL‐NOS	0.942 (0.570 ~ 1.558)	0.817		
Other T‐NHL	1.373 (0.560 ~ 3.364)	0.488		
Primary site		0.713		
Upper third of stomach	Reference			
Mid and low of stomach	1.395 (0.690 ~ 2.824)	0.354		
Overlapping lesion of stomach	1.441 (0.588 ~ 3.527)	0.424		
Stomach, NOS	1.476 (0.754 ~ 2.889)	0.256		
Ann Arbor stage (I/II vs. III/IV)	1.117 (0.869 ~ 1.435)	0.389		
Symptom (A vs. B)	0.944 (0.485 ~ 1.840)	0.867		
Chemotherapy (No/unknown vs. Yes)	0.540 (0.335 ~ 0.871)	0.012	0.605 (0.369 ~ 0.992)	0.046
Radiotherapy (No/unknown vs. Yes)	0.450 (0.194 ~ 1.040)	0.062	0.490 (0.211 ~ 1.135)	0.096
Surgery (No/unknown vs. Yes)	0.726 (0.372 ~ 1.419)	0.349		
Treatment modality		0.191		
Chemotherapy only	Reference			
Surgery/Radiotherapy only	0.345 (0.082 ~ 1.448)	0.146		
Chemotherapy plus Surgery	0.823 (0.375 ~ 1.802)	0.625		
Chemotherapy plus Radiotherapy	0.389 (0.135 ~ 1.120)	0.080		

Abbreviations: ALCL, anaplastic large cell lymphoma; PTCL, peripheral T‐cell lymphoma.

Moreover, we also investigated the impact of different treatment modalities on OS and CSS. Univariate and multivariable analyses were performed in 106 patients who received treatment. Compared with patients receiving chemotherapy, chemotherapy plus radiotherapy could significantly improve PG‐TCL patients' OS (Table 2 multivariate analysis II and Figure 3E). However, the benefit of chemotherapy plus radiotherapy for CSS was not prominent in comparison to chemotherapy only (Table 3 and Figure 4E).

### 3.4 Predictive nomogram for OS

**FIGURE 5:**
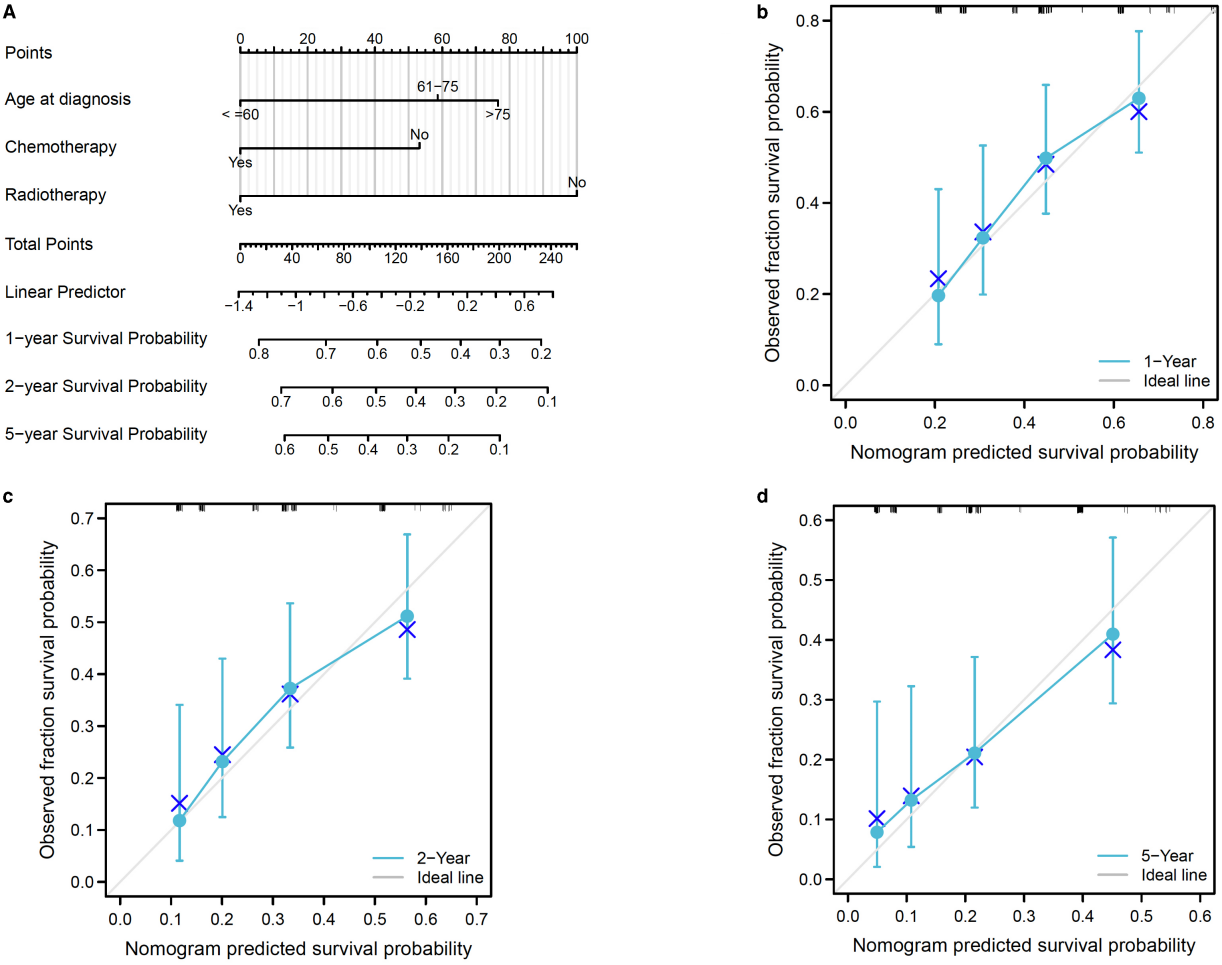
Predictive model for overall survival (OS) of patients with primary gastric T‐cell lymphoma (PG‐TCL) in SEER database. (A) Predictive nomogram for OS. (B–D) Calibration curves of the nomogram for 1‐, 2‐ and 5‐year OS.

Furthermore, a predictive nomogram model was constructed to predict OS for PG‐TCL based on independent risk factors of the Cox regression analysis, including age at diagnosis, radiotherapy, and chemotherapy. Figure 5A illustrated the OS nomogram at the 1‐, 2‐, and 5‐ years. The C‐index for OS was 0.655. Moreover, calibration curves (Figure 5B–D) showed that nomogram‐predicted values were almost consistent with actual observation values, suggesting the predictive accuracy of newly established nomograms.

### 3.5 Comparison of PG‐TCL and other histological types of PGL

**FIGURE 6:**
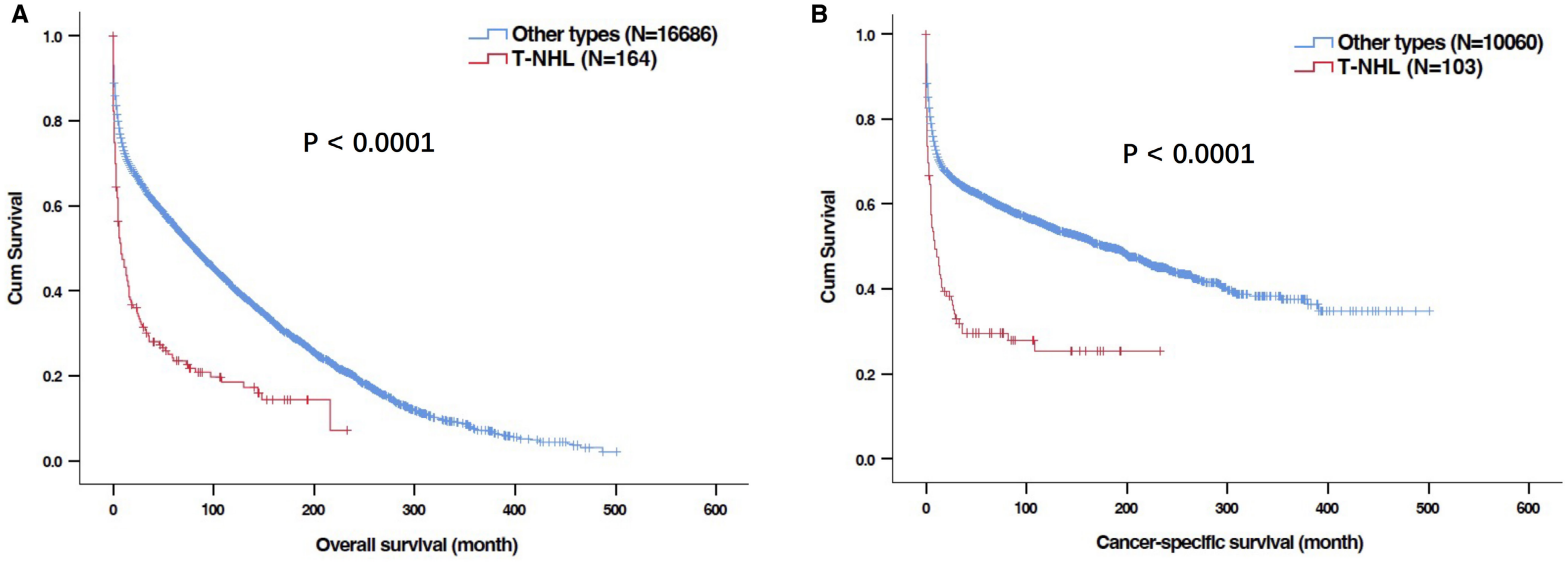
Kaplan–Meier curves for overall survival (OS) and cancer‐specific survival (CSS) in patients with primary gastric T‐cell lymphoma (PG‐TCL) and other histological types of lymphoma in SEER database. (A) OS; (B) CSS.

Then, we investigated the difference of clinical characteristics and prognosis between patients with PG‐TCL (*n* = 164) and other histological subtypes of PGL (*n* = 16,726) from SEER database. The distribution and percentage of different subtypes of PGL were shown in Figure S1 and Table S2. PG‐TCL only accounted for 0.97% of total PGL, which was far lower than primary gastric B‐cell lymphoma. Compared with other types of PGL, patients with PG‐TCL were characterized by younger age of diseases onset, male predominance, less White race, advanced disease stage, and more frequent presence of symptom B (Table 4). The OS and CSS of PG‐TCL patients were significantly worse than those of other types of PGL patients (median OS: 8.0 months vs 82.0 months, *P* < 0.001; median CSS: 9.0 months vs 78.0 months, *P* < 0.001; Figure 6A,B).

**TABLE 4:** Comparison of clinical characteristics of primary gastric lymphoma according to histological types

Characteristic	T‐cell gastric lymphoma (*N* = 164)	Other types of gastric lymphoma (*N* = 16,726)	
	*N* (%)	*N* (%)	*P* value
Age at diagnosis (years old)			0.004^a^
Mean ± SD	63.4 ± 17.6	67.4 ± 15.0	
Median (Range)	65 (8 ~ 93)	69 (0 ~ 105)	
Sex			<0.001
Male	113 (68.9)	8936 (53.4)	
Race			<0.001^b^
White	104 (63.4)	13,406 (80.2)	
Black/Other	59 (36.0)	3142 (18.8)	
Unknown	1 (0.6)	178 (1.1)	
Primary site			0.057
Upper third of stomach	27 (16.5)	1951(11.7)	
Mid and low of stomach	47 (28.7)	6341 (37.9)	
Overlapping lesion of stomach	17 (10.4)	1620 (9.7)	
Stomach, NOS	73 (44.5)	6814 (40.7)	
Ann Arbor stage			<0.001^b^
I/II	85 (51.8)	10,442 (63.4)	
III/IV	58 (34.4)	3319 (19.8)	
Unknown	21 (12.8)	2965 (17.8)	
Symptom			<0.001^b^
A	47 (28.7)	4244 (25.4)	
B	40 (24.4)	1676 (9.8)	
Unknown	77 (46.9)	10,806 (64.8)	
Chemotherapy			0.002
Yes	93 (56.7)	7450 (44.5)	
No/unknown	71 (43.3)	9276 (55.5)	
Radiotherapy			0.001
Yes	17 (10.4)	3422 (20.5)	
No/unknown	147 (89.6)	13,304 (79.5)	
Surgery			0.219
Yes	23 (14.0)	2998 (17.9)	
No/unknown	141 (86.0)	13,728 (82.1)	
Treatment modality			0.002
No treatment/unknown	58 (35.4)	5743 (34.3)	
Chemotherapy only	67 (40.9)	5049 (30.2)	
Surgery only	8 (4.9)	1448 (8.7)	
Radiotherapy only	4 (2.4)	1757 (10.5)	
Chemotherapy plus Surgery	14 (8.5)	1064 (6.4)	
Chemotherapy plus Radiotherapy	12 (7.3)	1179 (7.0)	
Surgery plus Radiotherapy	1 (0.6)	328 (2.0)	
Triple treatment	0 (0.0)	158 (0.9)	

^a^ *P* value for Student's *t*‐test.

^b^ Excluding “unknown” patients for statistics.

### 3.6 Comparison of PG‐TCL and non‐gastric T‐cell lymphoma

**FIGURE 7:**
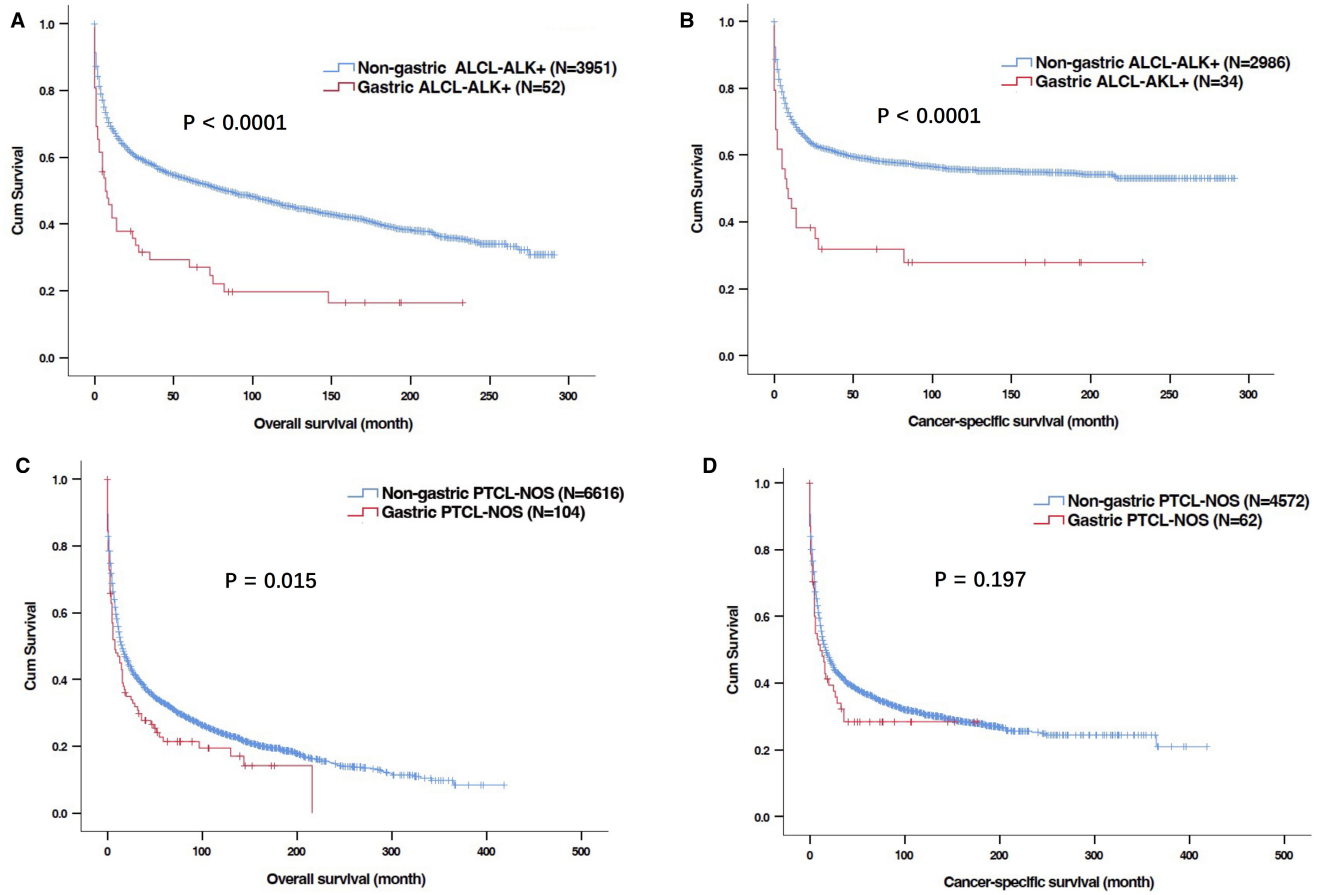
Kaplan–Meier curves for overall survival (OS) and cancer‐specific survival (CSS) in patients with primary gastric T‐cell lymphoma (PG‐TCL) and non‐gastric T‐cell lymphoma in SEER database. (A, B) OS (A) and CSS (B) of patient with primary gastric anaplastic large T‐cell lymphoma, anaplastic lymphoma kinase + (ALCL‐ALK+) and non‐gastric ALCL‐ALK (+); (C, D) OS (C) and CSS (D) of patient with primary gastric peripheral T‐cell lymphoma, not otherwise specified (PTCL‐NOS) and non‐gastric PTCL‐NOS.

As PTCL‐NOS and ALCL‐ALK+ were two major histological types of PG‐TCL, we further compared the clinical features and survival of patients between primary gastric PTCL‐NOS/ALCL‐ALK+ and non‐gastric PTCL‐NOS/ALCL‐ALK+. Primary gastric ALCL‐ALK+ patients exhibited older age of onset (Table 5), inferior OS (median OS: 7.0 months vs. 83.0 months, *P* < 0.001, Figure 7A) and CSS (median CSS: 8.0 months vs. not reached, *P* < 0.001, Figure 7B) compared with non‐gastric ALCL‐ALK+ patients. Primary gastric PTCL‐NOS patients were characterized by older age of onset, less White race, more focal involvement (Table 6), and shorter OS (median OS: 8.0 months vs. 16.0 months, *P* = 0.015, Figure 7C) than non‐gastric PTCL‐NOS patients, but no significant difference of CSS was found between primary gastric PTCL‐NOS and non‐gastric PTCL‐NOS patients (median OS: 11.0 months vs. 17.0 months, *P* = 0.197, Figure 7D).

**TABLE 5:** Comparison of clinical characteristics of primary gastric ALCL and non‐gastric ALCL patients

Characteristic	Primary gastric ALCL, ALK+ (*N* = 52)	Non‐gastric ALCL, ALK + (*N* = 3965)	*P* value
	*N* (%)	*N* (%)	
Age at diagnosis (years old)			0.005^a^
Mean ± SD	59.6 ± 17.6	51.1 ± 22.2	
Median (Range)	64.5 (8 ~ 93)	54 (0 ~ 103)	
Sex			0.244
Male	36 (69.2)	2431 (61.3)	
Race			0.315^b^
White	38 (73.1)	3156 (79.6)	
Black/Other	13 (25.0)	781 (19.7)	
Unknown	1 (1.9)	28 (0.7)	
Ann Arbor stage			0.944^b^
I/II	23 (44.2)	1761 (44.4)	
III/IV	23 (44.2)	1798 (45.3)	
Unknown	6 (11.5)	406 (10.2)	
Symptom			0.087^b^
A	5(9.6)	1020 (25.7)	
B	10(19.2)	821 (20.7)	
Unknown	37(71.2)	2124 (53.6)	
Chemotherapy			0.071
Yes	31 (59.6)	2817 (71.0)	
No/unknown	21 (40.4)	1148 (29.0)	
Radiotherapy			0.593
Yes	9 (17.3)	805 (20.3)	
No/unknown	43 (82.7)	3160 (79.7)	
Surgery			<0.001
Yes	6 (11.5)	1336 (33.7)	
No/unknown	46 (88.5)	2629 (66.3)	
Treatment modality			0.011
No treatment/unknown	17 (32.7)	610 (15.4)	
Chemotherapy only	21 (40.4)	1506 (38.0)	
Surgery only	1 (1.9)	303 (7.6)	
Radiotherapy only	2 (3.8)	137 (3.5)	
Chemotherapy plus Surgery	4 (7.7)	741 (18.7)	
Chemotherapy plus Radiotherapy	6 (11.5)	376 (9.5)	
Surgery plus Radiotherapy	1 (1.9)	98 (2.5)	
Triple treatment	0 (0.0)	194 (4.9)	

Abbreviation: ALCL, anaplastic large cell lymphoma.

^a^ *P* value for Wilcoxon signed‐rank test.

^b^ Excluding “unknown” patients for statistics.

**TABLE 6:** Comparison of clinical characteristics of primary gastric PTCL and non‐gastric PTCL patients

Characteristic	Primary gastric PTCL, NOS (*N* = 104)	Non‐gastric PTCL, NOS (*N* = 6690)	*P* value
	*N* (%)	*N* (%)	
Age at diagnosis (years old)			0.035^a^
Mean ± SD	65.6 ± 15.7	61.9 ± 17.7	
Median (Range)	68.5 (22 ~ 90)	64 (0 ~ 100)	
Sex			0.070
Male	71 (68.3)	3979 (59.5)	
Race			<0.001^b^
White	61 (58.7)	4958 (74.1)	
Black/Other	43 (41.3)	1651 (24.7)	
Unknown	0 (0.0)	81 (1.2)	
Ann Arbor stage			<0.001^b^
I/II	59 (56.7)	2094 (31.3)	
III/IV	33 (31.7)	3515 (52.5)	
Unknown	12 (11.5)	1081 (16.2)	
Symptom			0.672^b^
A	39 (37.50)	1953 (29.2)	
B	28 (26.9)	1261 (18.8)	
Unknown	37 (35.6)	3476 (52.0)	
Chemotherapy			<0.001
Yes	58(55.8)	5131(76.7)	
No/unknown	46(44.2)	1559(23.3)	
Radiotherapy			0.085
Yes	8 (7.7)	903 (13.5)	
No/unknown	96 (92.3)	5787 (86.5)	
Surgery			0.005
Yes	17 (16.3)	1939 (29.0)	
No/unknown	87 (83.7)	4751 (71.0)	
Treatment modality			<0.001
No treatment/unknown	37 (35.6)	974 (14.6)	
Chemotherapy only	42 (40.4)	3169 (47.4)	
Surgery only	7 (6.7)	399 (6.0)	
Radiotherapy only	2 (1.9)	122 (1.8)	
Chemotherapy plus Surgery	10 (9.6)	1245 (18.6)	
Chemotherapy plus Radiotherapy	6 (5.8)	486 (7.3)	
Surgery plus Radiotherapy	0 (0.0)	64 (1.0)	
Triple treatment	0 (0.0)	231 (3.5)	

Abbreviation: PTCL, peripheral T‐cell lymphoma.

^a^ *P* value for Student's *t*‐test.

^b^ Excluding “unknown” patients for statistics.

## 4 DISCUSSION

Given the rarity of PG‐TCL, clinical characteristics and prognosis have not been comprehensively studied yet. Large‐scale databases, such as SEER program, provided us the epidemiology and clinical features of PG‐TCL. To the best of our knowledge, this is the largest study about PG‐TCL to date. There were several observations obtained from the current study.

First, we reported the low age‐adjusted incidence rate of PG‐TCL with 0.0091 per 100,000 person‐years among US population for the first time. The incidence rate increased with age and males were more susceptible to PG‐TCL. In addition, this histological subtype was also rare among different types of PGL. In SEER database, PG‐TCL represented 0.97% of total PGL cases. In a previous study by Koch P, et al, PG‐TCLs occurred 1.3% (5/398) of total localized PGL (Ann Arbor Stages I and II) cases in Germany population,^15^ which was in line with our data.

Secondly, the current study demonstrated that PG‐TCL mainly occurred in the elderly and was more prevalent in males. The most common histological type of PG‐TCL in our study was PTCL‐NOS, followed by ALCL‐ALK+. These findings were consistent with previous reports from other institutions.^14^, ^16^, ^20^ The median OS was 8.0 months, and the 5‐year OS was 23.5% in our cohort. However, the 5‐year OS of PG‐TCL patients in Park et al.’s study^14^ and Kawamoto et al.’s study^16^ was about 40% and 55%, respectively, which was far higher than that of our cohort. The distinction in OS may be partially explained by different race and lower proportion of patients without receiving treatment in their studies. In terms of histological types of lymphoma, no difference in clinical features and survival was found among different types of PG‐TCL patients. However, when compared with other histological types of PGL cases (B‐cell lymphoma and Hodgkin's lymphoma), PG‐TCL cases exhibited male dominant, less White race, advanced disease stage, more frequent presence of symptom B as well as markedly poorer survival. With regards to primary sites, patients with primary gastric PTCL‐NOS/ALCL‐ALK+ had shorter OS than those with non‐gastric PTCL‐NOS/ALCL‐ALK+. Taken together, PG‐TCL patients had inferior survival and distinct clinical characteristics. More emerging treatments, for example, targeted therapy and immunotherapy, thereby warrant further development to overcome the poor prognosis of PG‐TCL.

Thirdly, independent survival factors of PG‐TCL patients were identified in the current study by log‐rank tests and Cox proportional hazards regressions. Age at diagnosis was the independent demographic factor and had an adverse impact on OS, which could be partly accounted for poorer performance status, more comorbidities, and higher incidence of toxicities of chemotherapy. On the other hand, the treatment modality provided to patients varied considerably in our cohort. Chemotherapy was still the first‐line treatment regime of PG‐TCL. More than half of the patients received chemotherapy. Multivariate regression analyses further indicated that chemotherapy was significantly associated with improved OS. There has been no consensus on standard chemotherapy regime for PG‐TCL yet. The chemotherapy regime CHOP, which consists of cyclophosphamide, doxorubicin, vincristine, and prednisolone, was most commonly used in Park et al.’s cohort.^14^ Surgical resection is often provided to patients with lymphoma for the purpose of obtaining biopsy tissue for pathological diagnosis as well as removing the primary tumor lesions or tumor debulking. The beneficial role of surgery for PGL has not been well defined. In the current study, use of surgery did not contribute a superior survival for patients with PG‐TCL, which was consistent with Huang et al's report that use of chemotherapy combined with surgery did not demonstrate survival benefits to PGL patients in comparison to chemotherapy alone.^21^ Nevertheless, a SEER population‐based study demonstrated that surgery contributed to better prognoses in primary gastric DLBCL patients.^10^ Moreover, our study verified radiotherapy as a favorable prognostic factor for PG‐TCL patients. When compared with chemotherapy alone, chemotherapy combined with radiotherapy favorably led to the enhancement of OS. This was inconsistent with the finding by Mehmet et al. that primary gastric DLBCL patients did not benefit from radiotherapy,^22^ which might be accounted for the different histology types.

Fourthly, as concerns statistical methodology, CSS was included as one of the endpoints of our study as it could directly reflect the influence of the disease on long‐term survival of patients and exhibit a more “dynamic” estimate of the risk of death over time than traditional survival rate. This study identified age at diagnosis and chemotherapy as the independent prognostic factors of CSS while radiotherapy might improve CSS of patients with PG‐TCL. Besides, from the CSS plot (Figure 2B), high risk of cancer‐specific death among PG‐TCL patients could be found in the first 2 years after diagnosis but became relatively steady after 2 years, indicating active follow‐up should be provided to those patients during the first 2 years after treatment. Furthermore, predictive nomogram of OS was developed by incorporating the independent risk factors in the present study, which could help us to precisely predict individual survival probability at certain time. However, it is also worth noting that CSS may be biased by misclassification of cause of death or informative censoring.^23^, ^24^ Therefore, the current consensus suggested that OS is generally preferable to CSS for calculating the marginal net survival for population‐based studies.^25^

There were some limitations to the present study. First of all, data selection bias may be inevitable due to the retrospective manner of the study. Next, in addition to the prognostic factors included in the current study, other factors, such as genetic mutation, performance status, chemotherapy regimens, specific dosage of radiation, different pathological types (ALCL‐ALK−), etc. also have an impact on survival. But the detailed information was unavailable to us from SEER database. Additionally, whether the patients did not receive therapy, or the treatment information was unknown could not be discriminated based on the data extracted from SEER database. The insufficient information limited us for further investigating the effects of these important components on prognosis. Moreover, there has been no consensus about the definition of PGL so far. In the strict sense, only patients with Ann Arbor Stages I and II would be included in our study according to the original definition of PGL.^1^ Nevertheless, other researchers widened this definition later, in which the involvement of lesions was not limited to stomach and regional lymphadenopathy.^2^, ^3^, ^4^ Due to small sample size of localized PG‐TCL patients (Ann Arbor Stages I and II), patients with Ann Arbor Stages III and IV were also included the current study based on the widened definition of PGL.^2^, ^3^, ^4^ The clinical outcomes and prognosis of localized PG‐TCL patients should be explored with larger samples size in the future. Finally, it is worthwhile that due to low incidence of this neoplasm, the patients undergoing surgery or radiotherapy were limited in the present study. Hence, the results of univariate and multivariate analyses regarding to treatment modality need to be interpreted with caution.

Taken together, this is the largest and first population‐based retrospective study about PG‐TCL, which consisted of 164 patients from SEER database and indicated distinct clinical characteristics and inferior prognosis of PG‐TCL with low incidence in US population. Our study suggested that chemotherapy was the main treatment option for PG‐TCL, and the addition of radiotherapy could improve patients' survivals, which could improve clinicians' knowledge about the treatment of PG‐TCL. The predictable nomogram facilitated hematologists to estimate prognosis and establish personal follow‐up strategy.

## AUTHOR CONTRIBUTIONS

Minyue Zhang and Fei Xiao contributed the conception of the study and wrote the paper. Meisi Lin, Minyue Zhang, and Mengping Chen collected and analyzed the data. Jian Hou designed the study and Honghui Huang contributed to the revision of the manuscript. All authors approved the final manuscript.

## FUNDING INFORMATION

This work was supported by Science and Technology Development Foundation of Shanghai Pudong New Area Health and Family Planning Commission (PW2015E‐1).

## CONFLICT OF INTEREST

No conflicts of interest.

## ETHICS STATEMENT

The patient information is not contained in the SEER program. Institutional review board approval and informed consent from the patients were not required.

## Supporting information


Tables S1‐S2

Figure S1

## Data Availability

Data were publicly accessible which can be obtained in the SEER database.
