# Supplementary material for: The epidemiology and prognosis of patients with primary gastric T‐cell lymphoma in the SEER program
Source: Cancer Med. 2022 Jun 13;12(1):84–98. doi: 10.1002/cam4.4936 (PMC9844593; doi:10.1002/cam4.4936)
Supplement: Supplementary file 1 — Tables S1‐S2 [file CAM4-12-84-s001.docx]

**Supp Table 1. Comparison of clinical characteristics of primary gastric T-cell lymphoma according to histologic types**

| **Characteristic** | **PTCL, NOS (N=104)** | **ALCL, ALK+ (N=52)** | **Other T-NHL (N=8)** | ***P* value** |
| --- | --- | --- | --- | --- |
|  | **N (%)** | **N (%)** | **N (%)** |  |
| Age at diagnosis (years old) |  |  |  |  |
| Mean±SD | 65.6±15.7 | 59.6±17.6 | 58.4±18.0 | 0.232^#^ |
| Median (range) | 68.5 (22~90) | 64.5 (8~93) | 62 (29~84) |  |
| Sex |  |  |  |  |
| Male | 71 (68.3) | 36 (69.2) | 6 (75.0) | 1.000 |
| Female | 33 (31.7) | 16 (30.8) | 2 (25.0) |  |
| Race |  |  |  |  |
| White | 61 (58.7) | 38 (73.1) | 5 (62.5) | 0.180* |
| Black | 30 (28.8) | 7 (13.5) | 3 (37.5) |  |
| Other | 13 (12.5) | 6 (11.5) | 0 (0) |  |
| Unknown | 0 (0) | 1 (1.9) | 0 (0) |  |
| Primary site |  |  |  |  |
| Upper third of stomach | 18 (17.3) | 8 (15.4) | 1 (12.5) | 0.561 |
| Mid and low of stomach | 27 (26) | 17 (32.7) | 3 (37.5) |  |
| Overlapping lesion of stomach | 8 (7.7) | 8 (15.4) | 1 (12.5) |  |
| Stomach, NOS | 51 (49) | 19 (36.5) | 3 (37.5) |  |
| Ann Arbor stage |  |  |  |  |
| I/II | 59 (56.7) | 23 (44.2) | 3 (37.5) | 0.306* |
| III/IV | 33 (31.8) | 23 (44.2) | 2 (25.0) |  |
| Unknown | 12 (11.5) | 6 (11.6) | 3 (37.5) |  |
| Symptom |  |  |  |  |
| A | 39 (31.7) | 5 (9.6) | 3 (37.5) | 0.175* |
| B | 28 (26.7) | 10 (19.2) | 2 (25.0) |  |
| Unknown | 37 (36.2) | 37 (71.2) | 3 (37.5) |  |
| Chemotherapy |  |  |  |  |
| Yes | 58 (55.8) | 31 (59.6) | 4 (50.0) | 0.824 |
| No/unknown | 46 (44.2) | 21 (40.4) | 4 (50.0) |  |
| Radiotherapy |  |  |  |  |
| Yes | 8 (7.7) | 9 (17.3) | 0 (0.0) | 0.138 |
| None/unknown | 96 (92.3) | 43 (82.7) | 8 (100.0) |  |
| Surgery |  |  |  |  |
| Yes | 17 (16.3) | 6 (11.5) | 0 (0.0) | 0.497 |
| Not recommended/unknown | 87 (83.7) | 46 (88.5) | 8 (100.0) |  |
| Treatment modality |  |  |  |  |
| No treatment received | 37 (35.6) | 17 (32.7) | 4 (50.0) | 0.182 |
| Chemotherapy only | 42 (40.4) | 21 (40.4) | 4 (50.0) |  |
| Radiotherapy/Surgery only | 9 (8.7) | 3 (5.8) | 0 (0.0) |  |
| Combined treatment | 16 (15.4) | 11 (21.2) | 0 (0.0) |  |

# P value for Kruskal-Wallis test; * Excluding “unknown” patients for statistics

Abbreviation: ALCL, anaplastic large cell lymphoma; PTCL, peripheral T-cell lymphoma; T-NHL, T-cell non-Hodgkin lymphoma

**Supp Table 2. Composition of different histological subtypes of primary gastric lymphoma**

| **Subtype of lymphoma** | **N** | **Percentage (%)** |
| --- | --- | --- |
| Hodgkin lymphoma | 43 | 0.25 |
| Mature B-cell neoplasms |  |  |
| Mantle-cell lymphoma | 214 | 1.26 |
| Malignant lymphoma, small B lymphocytic, NOS | 334 | 1.97 |
| Lymphoplasmacytic lymphoma | 46 | 0.27 |
| Diffuse large B-cell, NOS | 7483 | 44.05 |
| Burkitt lymphoma | 183 | 1.08 |
| Mucosa associated lymphoid tissue lymphoma | 5985 | 35.23 |
| Follicular lymphoma | 332 | 1.95 |
| Plasmablastic lymphoma | 21 | 0.12 |
| Malignant lymphoma, mixed small & large cell, diffuse | 160 | 0.94 |
| Primary cutaneous follicle centre lymphoma | 1 | 0.01 |
| Mature T-cell and NK-cell neoplasms |  |  |
| Peripheral T-cell lymphoma, NOS | 104 | 0.61 |
| Angioimmunoblastic T-cell lymphoma | 2 | 0.01 |
| Anaplastic large cell lymphoma, ALK+ | 52 | 0.31 |
| Enteropathy-associated T-cell lymphoma | 2 | 0.01 |
| Extranodal NK-/T-cell lymphoma, nasal type | 4 | 0.02 |
| Precursor B lymphoblastic lymphoma | 3 | 0.02 |
| Non-Hodgkin’s lymphoma, NOS | 912 | 5.37 |
| Composite Hodgkin lymphoma and non-Hodgkin’s lymphoma | 6 | 0.04 |
| Malignant lymphoma | 1003 | 5.90 |
| Total | 16890 | 100.00 |
