# Supplementary figures and images for: The epidemiology and prognosis of patients with primary gastric T‐cell lymphoma in the SEER program
Source: Cancer Med. 2022 Jun 13;12(1):84–98. doi: 10.1002/cam4.4936 (PMC9844593; doi:10.1002/cam4.4936)

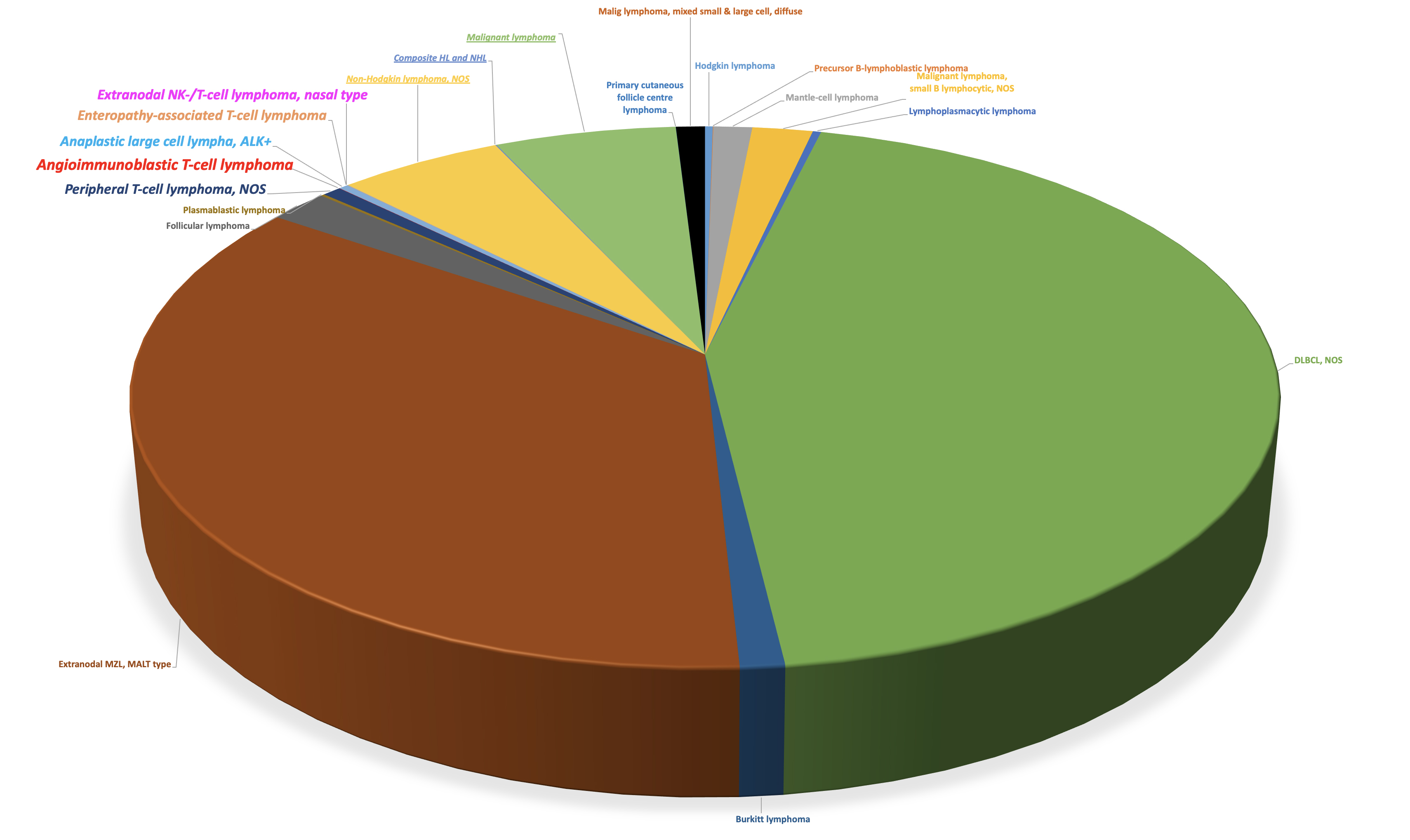

Supplement: Supplementary file 2 — Figure S1 [file CAM4-12-84-s002.jpg]
